# Supplementary material for: Are carbon emissions trading and green financial instruments synergistic? -Comprehensive quantitative research based on content analysis
Source: PLoS One. 2024 Mar 7;19(3):e0298601. doi: 10.1371/journal.pone.0298601 (PMC10919601; doi:10.1371/journal.pone.0298601)
Supplement: S1 Data — (ZIP) [file pone.0298601.s001.zip › Data/地级市Z_F工作报告.zip!/║╙─╧/╓ú╓▌╩╨/└┤╫╘╣1⁄2╓┌║┼í╛┬φ┐╦╩2╛▌═°í┐_2007.docx]

各位代表：

　　现在，我代表市人民政府，向大会作工作报告，请予审议，并请各位政协委员和列席人员提出意见。

　　2006年政府工作回顾

　　2006年，市人民政府在省委、省政府和市委的正确领导下，紧紧依靠全市人民，全面落实科学发展观，加快构建和谐社会，认真贯彻党的十六大和十六届三中、四中、五中、六中全会精神和各项方针、政策，按照“加快发展、维护稳定、为民谋利”的总体要求，以实施跨越式发展八项重点工程为抓手，锐意创新，真抓实干，圆满完成了市十二届人大三次会议确定的各项目标任务，全市经济建设、政治建设、文化建设和社会建设都取得了新成绩，实现了“十一五”规划和跨越式发展三年行动计划的良好开局。

　　初步统计，全市实现生产总值2001.5亿元，增长15.7%；地方财政收入202.4亿元，增长34%；规模以上工业企业实现销售收入2291.6亿元，增长38.4%；全社会固定资产投资新增211.9亿元，达到1031.9亿元，增长25.8%；利用域外资金248.34亿元，增长92.1%；社会消费品零售总额822.2亿元，增长16.3%；居民消费价格上涨1.4%；城镇居民人均可支配收入达到11822元，增长11.1%；农民人均纯收入达到5559元，增长16.4%。非公有制经济完成增加值1100亿元，增长23%，占全市生产总值的比重达到55%。

　　??工业经济运行良好，质量和效益显著提高。坚持扶优扶强，加快结构调整，工业经济质量和效益同步增长。全部工业增加值完成944亿元，增长19.7%，其中规模以上工业增加值完成723亿元，增长23%；实现利润213亿元，增长85.2%。“工业项目建设年”活动成效显著，工业项目完成投资378.9亿元，增长40%；富耐克超硬材料公司等企业的110个项目竣工投产。扶优扶强工程强力推进，54个项目开工建设，48户重点企业实现销售收入507亿元，增长32%；销售收入超亿元企业新增231户，超10亿元企业达到29户，宇通集团、中铝河南分公司2户企业首次突破百亿元。汽车及装备制造业、铝工业、能源工业、食品工业等优势产业的竞争力进一步增强，三全、宇通等6户企业的商标成为全国驰名商标。产业园区建设速度加快，服装、食品、铝加工、耐材等品牌园区、特色园区正在形成，全市33个重点工业园区完成工业增加值348亿元。

　　??农村经济持续增长，新农村建设进展顺利。狠抓各项支农惠农政策落实，农业结构和布局进一步优化，现代农业建设成效明显，新农村建设扎实推进，农业农村呈现持续发展的良好态势。全市农林牧渔业增加值77.1亿元，增长6.6%；粮食总产达164.9万吨；畜牧业、水产业得到了较快发展。农业产业化经营大力推进，18个现代农业示范园区已初具规模。市级以上重点龙头企业达181家，新发展农民专业合作社160个，全市农产品及深加工企业完成出口创汇1.5亿美元，增长160.8%。切实加大对农业的投入，全年安排财政支农资金6.9亿元，增长28.5%，对种粮农民实行粮食直补、良种补贴、农机具购置补贴、农资综合补贴等补贴措施全部落实到位，共补贴资金1.2亿元。新建改建农村公路2790公里，在全省率先实现村村通油路。新增农村户用沼气池4万座、集雨水窖1万个，解决17万人安全饮水问题，巩固和解决1万贫困人口脱贫。实施“百村示范、千村整治”工程，选派千名机关干部驻村帮助示范村开展工作，编制了示范村村庄建设规划及产业发展规划，1000个整治村中已有616个村的村容村貌整治取得初步成效。

　　??第三产业健康成长，现代服务业快速发展。传统服务业发展水平不断提升，麦德龙、易初莲花等国内外知名商业企业继续在郑州扩大投资，丹尼斯、正道花园等商业企业进一步做大做活。专业交易市场的辐射力进一步增强，12个批发市场交易园区已基本形成。“万村千乡市场工程”全面启动，我市被确定为全省首批整县推进的试点市。现代物流业快速发展，郑州铁路集装箱中心站、郑州国家干线公路物流港等项目开工建设，公路港、铁路港、航空港、信息港“四位一体”的物流体系日趋完善，豫鑫物流、市交运集团和河南长通分别被评为4A级和3A级物流企业。会展业管理经营逐步迈向市场化、国际化，全国制药机械博览会、第12届郑交会、首届豫商大会等大型展会在我市成功举办。重点文化产业项目建设进展顺利，炎黄二帝塑像工程项目建设基本完成，升达艺术馆改扩建工程动工，《风中少林》入选国家十大舞台艺术精品剧目和中宣部十大优秀作品，《禅宗?少林》音乐大典成功上演。新闻出版产业发展迅速，郑州印刷包装工业园建设取得实质性进展。旅游业健康发展，旅游总收入230.5亿元，增长28.56%。房地产业平稳发展，全年完成增加值62.5亿元，增长6.6%。区域性金融中心建设积极推进，金融机构存款余额达3560亿元，增长14.4%。郑州商品交易所交易额实现31809.6亿元，新推出PTA期货交易品种。保险、中介等服务业快速发展。

　　??县域经济发展迅猛，综合实力显著增强。不断完善落实加快县域经济发展的政策措施，拿出3000万元资金表彰奖励30个先进乡(镇)，推动县域经济快速发展。各县(市)结合本地实际，以发展特色工业园区和产业集群区为重点，积极培育特色经济和块状经济，形成了一批具有带动力和竞争力的县域特色工业园区。初步核算，全市县域实现生产总值1113亿元，占全市经济总量的55.6%，6县(市)生产总值均超过百亿元；地方财政收入49.1亿元，占全市地方财政收入的24.3%，巩义市达到12.64亿元，登封市首次突破10亿元；全社会固定资产投资完成563.7亿元，占全市投资总额的54.6%；城镇化率达到37%。有5个县(市)经济实力位列全省十强。巩义市在全国百强县位次比上年前移6位，新郑市首次跻身全国百强县行列。

　　??城市建设力度不断加大，城市管理水平逐步提高。中心城区保护改造继续得到加强。新修、扩建道路13条，伏牛路、北环文化路立交、未来大道等续建项目已完工，南三环东段、郑密路等工程进展顺利。中原路、花园路等精品街综合整治顺利推进；完成雨污水工程改造45项，铺设雨污水管涵63公里；新开、优化公交线路47条，新购运营车辆357台；新建公园、游园30个，中心城区新增绿地300.3万平方米，全市绿化覆盖率达35.1%。天然气战略储配站、郑东热源厂和郑热三期配套管网相继建成投产使用，提高了市区供气、供热能力。城中村改造有序推进。

　　城市管理水平进一步提高，数字化城市管理系统建设全线展开，出租汽车、户外广告整治取得新进展。荣获国家卫生城市、国家园林城市称号。

　　郑东新区建设步伐加快。累计完成投资368.8亿元，引进项目196个，开工项目167个，在建和建成房屋面积1464万平方米，入驻人口13万人。基础设施逐步完善，建设道路197条(段)，东西运河、南北运河开挖工程竣工通航，开通了18条公交线路，绿化面积达700万平方米。重点区域建设进展顺利。中央商务区内、外环高层开工55栋，35栋主体结顶，郑州国际会展中心通过国家“鲁班奖”验收，河南艺术中心主体工程已全部封顶，会展宾馆开工建设；龙湖南区入驻项目25个，22个开工建设；商住物流区入驻项目70个，61个开工建设。

　　积极推进中原城市群建设与发展，郑开大道正式开通，郑汴产业带总体规划编制完成，郑汴协调发展机制基本建立，郑汴一体化取得了实质性进展。中小城市和小城镇建设实现新的突破。科学规划，合理布局，不断加大投入力度，重点支持市级重点镇建设，全市31个重点镇基础设施投资项目开工105项，完成投资3亿多元。

　　??各项改革稳步推进，对外开放水平不断提高。国有企业改革取得新进展，20户国有企业已完成改制。以中小学校移交为重点的企业主辅分离工作全部完成。白鸽股份完成股改和资产重组，市商业银行、百瑞信托、中小企业担保公司增资扩股进展顺利。国有资本经营预算制度和经营业绩考核体系逐步健全，国有资产实现依法监管。上市融资成效明显，思念公司成功在新加坡主板上市，豫联集团、永通特钢成功增资，郑州燃气转香港主板上市工作顺利推进。事业单位改革稳步推进，粮食流通体制改革继续深化，农村综合改革顺利实施。行政审批制度改革步伐加快，全市所有行政许可事项实现了网上统一受理和即时办结。机关事业单位工资制度改革和行政单位清理规范津贴补贴工作顺利实施，公务员登记工作基本完成。

　　对外开放进一步扩大。改进招商方式，重点实施节会招商、以商招商等多种招商手段，利用丙戌年黄帝故里拜祖大典、第二届世界传统武术节等大型活动，积极开展招商引资，取得显著成绩。全年引进市外境内资金200.2亿元，增长95.7%。全年新增外商投资企业135家；实际利用外资6.1亿美元，增长83%。开放型经济发展实现新突破，全市进出口总额达到17.3亿美元，增长55%，其中出口12.1亿美元，增长56.1%。获得对外经济合作经营权企业累计达32家，占全省的1／3。对外经济技术交流与合作全方位拓展，区域经济协作与联络明显加强。

　　??资源节约工作扎实推进，环境保护得到加强。继续开展煤炭、铝土矿和耐火材料资源整合，资源集中度和利用率明显提高。继续开展空心村、工矿废弃地、黏土砖瓦窑场整治，复垦土地5227亩。加强对重点领域、高耗能行业、重点用能单位的节能管理，加快淘汰落后产能，关停了一批小火电、小耐材和普通立窑水泥厂，全年万元生产总值能耗下降5%左右。资源节约和清洁生产成效初显，循环经济试点顺利实施，全年化学需氧量和二氧化硫排放量分别下降1.7%和3.3%。重点行业、重点区域和重点流域环境综合整治得到加强，省定375家污染严重的企业全部关闭到位，新密、巩义耐火材料重点整治成效明显，东风渠、熊儿河、金水河截污清淤工程基本完成，贾鲁河河道治理疏浚工程已经完成。王新庄污水处理厂升级改造和马头岗、上街污水处理厂等项目正在积极推进，新郑、中牟等5个县(市)污水处理厂已进水调试。城市生活污水集中处理率和工业固体废物综合利用率分别达到56.1%和66%，全市医疗废物集中处置率达到99%以上。生态环境质量继续改善，完成森林生态城工程造林8500公顷，市区空气质量二级以上天数达306天。全市饮用水源地水质达标率为100%。国家环保模范城创建已通过国家技术评估，创建全国绿化模范城市工作全面启动。

　　??各项社会事业全面进步，构建和谐社会取得新进展。科技自主创新工程进展态势良好，科技进步对国民经济的贡献率达到51%。教育事业快速发展，义务教育均衡发展步伐加快，市区新建、改扩建中小学10所。全面免除了城市低保家庭和农村学生义务教育阶段学杂费，全市9.9万名中小学贫困生得到救助，全面启动了农村寄宿制学校建设工程，农村中小学危房改造长效机制初步形成。职业教育发展进一步提速，各类职业技术培训达到60万人次。公共卫生服务体系不断完善，7所示范乡镇卫生院建成并投入使用，改造了32所乡镇卫生院和一批县级医院、妇幼保健院和中医院。全市农村新型合作医疗制度全面推行，参合率达到90.75%。实施了食品放心工程，农产品质量安全检验检测体系逐步完善，严格市场准入和执法，食品药品质量安全工作得到加强。人口和计划生育工作继续加强，人口自然增长率控制在4.5‰。公共文化基础设施建设取得新进展，商城遗址公园、大河村原始文化生态园等立项筹建工作进展顺利。“扫黄打非”工作取得显著成效，全市出版物市场得到有效整治。社区基础设施建设不断加强，建设和谐社区取得实效。首届农民运动会成功举办，郑州籍运动员在多哈亚运会上夺得3枚金牌，群众性文化体育活动广泛开展。坚持和完善政府与工会联席会议制度，充分发挥工会、共青团、妇联等群团组织的桥梁和纽带作用，切实维护广大职工群众的利益，切实保障妇女、未成年人、老年人和残疾人的合法权益。民兵预备役建设和优抚安置工作得到加强，双拥共建、国防教育、人民防空工作取得新成效。

　　就业再就业工作成效显著，实现城镇就业再就业12.5万人，农村劳动力转移就业13.9万人，对农村劳动力进行引导性培训15.8万人。社会保障体系进一步完善，全年各级财政投入社会保障资金13.6亿元，全市新增企业养老保险参保职工7.9万人，新增基本医疗保险参保人员8万人，为4.2万名市属国有破产企业和困难企业退休人员办理了大病统筹医疗保险，农民工工伤保险工作扎实推进，城乡低保标准进一步提高。社会救助得到加强，扶贫开发和救灾救济工作扎实有效。市区新建1所社会福利院，市儿童福利院正在加紧建设中，农村新建、扩建敬老院31所。新开工经济适用住房89万平方米，廉租住房覆盖率达到符合条件“双困家庭”100%。

　　?民主与法制建设继续加强，社会大局安定有序。自觉接受人民代表大会及其常委会监督，接受人民政协民主监督，认真听取民主党派、工商联、无党派人士、各人民团体的意见。全年办复人大代表议案、建议和政协委员提案1566件。全面推进依法治市，建立权责明确、行为规范、监督有效、保障有力的行政执法体制，提请审议地方性法规5件，制定规章10件。大力推进政务公开，政府新闻发言人制度和网上审批逐步完善。进一步完善政府重大决策机制，健全重大决策专家论证、决策评估制度，政府决策的科学性和透明度进一步提高。认真落实党风廉政建设责任制各项任务，政府廉政建设和反腐败工作取得新成效。严格落实安全生产责任制，建立了市、县、乡、村、企业“五级监管网络”，对重点行业和领域进行全面检查和专项整治，安全生产形势良好。深入贯彻《信访条例》，畅通信访渠道，加大督查督办力度，认真解决长期信访积案和重复信访问题，妥善化解矛盾，群体性突发事件大幅减少。继续深化“两严一创”活动，深入开展命案攻坚，不断完善社会治安防控体系，全面加强社会治安综合治理，“五五”普法顺利启动，社会治安持续稳定。

　　向群众承诺的“十件实事”，件件得到落实，一些社会热点、难点问题得到有效解决。

　　广播电视、统计、审计、物价、外事、侨务、对台事务、民族宗教、社会科学、地方志、档案管理、气象和地震监测等工作都取得了新的成绩。

　　回顾一年来的政府工作，我们深深体会到，我市经济社会能够实现又快又好的发展，得益于我们认真坚持和把握了以下几个方面：一是坚定不移地贯彻落实科学发展观。始终坚持“五个统筹”，不断加大对薄弱环节的投入，加快对外开放步伐，加强节能降耗和生态环保工作，大力发展循环经济，强力推进县域经济，实现了经济社会全面协调可持续发展。二是坚定不移地贯彻落实中央宏观调控政策。正确处理宏观调控和加快发展的关系，坚持“有保有压”的原则，不断优化投资结构，把国家加强薄弱环节的政策与郑州发展的重点有机结合起来，在发展中顺应调控，在调控中谋求发展。三是坚定不移地把握正确的发展思路。准确把握市情，理清发展思路，年初制定并出台了经济社会跨越式发展三年行动计划，规划并实施了八项重点工程，加大结构调整和重大项目建设力度，使经济结构调整和产业优化升级步伐明显加快。四是坚定不移地推进体制机制创新。继续以国有企业改革和非公有制经济发展为重点，全面深化和推进粮食流通体制、投融资体制、农村信用社等重点领域的改革，着力解决经济发展中的深层次矛盾，市场在资源配置中的基础性作用和微观经济的活力明显增强。五是坚定不移地优化发展环境。大力改善投资环境，开展了“整治建设环境和企业周边环境”等专项治理，对久拖不决的外商投诉案件实行领导包案，着力解决损害经济发展的突出问题，加强重点建设项目效能督查，为外商投资创造统一、稳定、透明的法律环境、政策环境。

　　各位代表，上述成绩的取得，是省委、省政府和市委正确领导的结果，是市人大、市政协积极支持的结果，也是全市广大干部群众共同奋斗的结果。在此，我代表市人民政府，向给予政府工作大力支持的人大代表和政协委员，向工作在各条战线上的工人、农民、知识分子和干部，向驻郑人民解放军、武警官兵、公安政法干警，向各民主党派、工商联、无党派人士、人民团体和各界人士，表示崇高的敬意！向关心和支持郑州发展的香港特别行政区同胞、澳门特别行政区同胞、台湾同胞、海外侨胞和国际友人，表示诚挚的感谢！

　　在肯定成绩的同时，我们也要清醒地看到经济社会发展中存在的突出矛盾和问题：我市经济结构性矛盾依然存在，投资和产业结构不尽合理，资金、土地、能耗、环境保护等制约我市经济发展的瓶颈问题仍然突出；科技创新能力不足，高新技术产业比重较小；农民就医难、增收缓慢仍然是迫切需要解决的问题，新农村建设任务艰巨；低收入群体在住房、医疗、教育等方面还有不少困难，构建和谐社会的任务依然艰巨；利用外资规模偏小，经济外向度不高；城市管理水平有待进一步提高，创建文明城市的任务仍然艰巨；节能降耗任务不容乐观，完成降耗减排目标的难度将越来越大；一些政府工作人员服务意识不强、效率不高，政府廉政勤政建设有待进一步加强。这些问题，都需要在今后的工作中切实加以改进和解决。

　　2007年政府工作的基本要求和主要目标

　　今年政府工作的基本要求：以邓小平理论和“三个代表”重要思想为指导，认真贯彻党的十六大和十六届三中、四中、五中、六中全会及省八次党代会、市九次党代会精神，以科学发展观统领经济社会发展全局，加快构建社会主义和谐社会，按照“加快发展、维护稳定、为民谋利”的总体要求，围绕经济社会跨越式发展奋斗目标，进一步加快工业化、城市化和农业现代化进程，着力调整经济结构和转变增长方式，着力加强资源节约和环境保护，着力推进改革开放和自主创新，着力促进社会发展和解决民生问题，促进全市经济社会又好又快发展，奋力实现经济发展新跨越，奋力实现文化发展新跨越，加快建设文化郑州、生态郑州、平安郑州、和谐郑州。

　　今年我市经济社会发展的主要目标：生产总值增长13%；地方财政收入增长15%；全社会固定资产投资增长23%；社会消费品零售总额增长14%；实际利用外商直接投资增长20%；居民消费价格总水平涨幅在3%以内；城镇居民人均可支配收入增长8%；农民人均纯收入增长8%；人口自然增长率控制在6‰以下；城镇登记失业率控制在4%以内；单位生产总值能耗降低5%，化学需氧量减少4%，二氧化硫排放量减少4.2%。

　　为全面完成上述目标任务，我们必须进一步解放思想，转变观念，认真把握好以下几个重要方面，切实把经济社会发展转到科学发展轨道：

　　一是加快经济结构调整和经济增长方式转变，推进二、三产业双重升级。继续推进经济结构战略性调整，处理好投资和消费的关系，在扩大投资规模的同时，进一步扩大内需，不断增强消费对我市经济增长的拉动作用。不断优化产业结构，大力发展先进制造业，大力发展现代服务业，提高经济增长的质量和效益，实现更高水平和更大规模的发展。

　　二是努力提高自主创新能力，加快建设创新型城市。坚持把增强自主创新能力作为发展的战略基点和调整产业结构、转变增长方式的中心环节。确立企业在技术创新中的主体地位，支持科研机构和企业开展核心技术和关键技术攻关，加快重点产业技术改造和升级。合理配置科技资源，充分激活创新要素的政策调控体系，加快体制创新、管理创新、科技创新，推动引进消化吸收再创新，加快建设创新型城市。

　　三是统筹城乡协调发展，推动和谐社会建设。坚持以人为本，更加关注民生问题，促进社会公平正义，促进城乡协调发展。进一步巩固和加强农业基础，全面发展农村经济，逐步建立工农协调发展的机制。坚持把扩大就业放在经济社会发展更加突出的位置，进一步完善收入分配制度，理顺收入分配关系，不断健全覆盖城乡居民的社会保障和社会救助体系，建立健全基本公共服务和社会管理体系，保持社会安定有序。

　　四是加强资源能源节约和环境保护，促进可持续发展。把资源能源节约和环境保护放在更加突出的位置，认真落实资源能源节约和环境保护基本国策，加快形成有效的资源开发和生态补偿机制，大力发展循环经济，以解决危害群众健康和影响可持续发展的环境问题为重点，加强环境污染治理和生态建设，切实做到节约发展、清洁发展、安全发展和可持续发展。

　　五是继续深化改革，进一步扩大对外开放。以提高竞争力和控制力为重点深化国有企业改革，以转变政府职能为重点加快行政管理、投资、财税体制等改革，以完善金融企业法人治理结构和优化金融结构为重点深化金融体制改革，以建立社会公平保障体系为目标加快社会领域体制改革。加快调整和完善对外经济发展模式，以引进先进技术、先进管理和海外智力为重点，提高对外贸易和利用外资质量和水平。

　　2007年的主要工作

　　今年是我市实现经济社会跨越式发展十分关键的一年。我市经济社会诸多方面发生了深刻变化，进入了全面提升产业层次、加快经济转型、促进统筹协调发展的新阶段，围绕加快建设现代物流中心、区域性金融中心，提升全国区域中心城市地位发展目标，着力做好以下十个方面的工作。

　　一、进一步加快结构调整，不断增强工业发展竞争力

　　坚持走新型工业化道路，推动工业经济快速增长。全部工业投入突破500亿元，规模以上工业销售收入达到2800亿元。以促进工业结构优化升级和经济增长方式转变为重点，做强做大汽车工业、装备制造业、铝工业、能源工业、重化工业、农副产品精深加工和生物化工等优势产业，大力发展超硬材料、电子信息、生物医药、光机电等高新技术产业，改造提升纺织、服装、建材等传统产业。充分利用国家振兴中部老工业基地有关政策，积极争取项目和资金，促进我市老工业基地改造升级。继续推进扶优扶强工程，完善重点企业培育机制，加大财政支持力度，鼓励宇通、豫联、三全等企业发展壮大，提升龙头骨干企业和优势企业的核心竞争力，力争年销售收入超百亿元企业达到3户。大力推进产业集群发展，突出抓好品牌园区、特色园区、重点园区建设。实施品牌发展战略，力争再创一批国家级名牌产品和在国际上有影响的知名品牌。

　　二、充分发挥比较优势，加快现代服务业发展

　　积极推进第三产业发展，提升现代服务业发展水平。大力发展连锁经营、物流配送、电子商务等现代流通方式，加快二七商圈、碧沙岗商圈的产业升级。大力发展现代物流业，加快圃田物流园区等物流基础设施建设，推进郑州铁路集装箱中心站、郑州国家干线公路物流港、郑州国际航空保税物流中心等重点物流项目建设，构建铁路、公路、航空、信息一体化物流体系；支持重点物流企业做大做强，努力培育一批大型现代物流企业集团。推动会展业加快升级，不断完善会展硬件设施，提高办展办会水平。认真筹办好第二届中部贸易投资博览会、郑交会等重点展会。打造商都文化苑、炎黄广场等旅游精品景区，培育郑汴洛、黄帝游、沿黄文化游等旅游精品线路，大力发展具有中原特色的文化旅游业。

　　三、大力发展现代农业，扎实推进新农村建设

　　把发展现代农业作为推进社会主义新农村建设的着力点，用现代物质条件装备农业，用现代科学技术改造农业，用现代发展理念引领农业，提高土地产出率、资源利用率和农业劳动生产率。大力发展优质、高效、专用农作物，逐步建立起各类具有区域化优势的产业化生产基地。大力发展以石榴为主的百里邙岭林果产业带。培育壮大农业产业化经营龙头企业，力争市级重点龙头企业突破200家。推进现代农业示范园区建设，力争有3个园区达到全省一流水平。加强农业科技推广和农业标准化体系建设，加快出口创汇农业发展，大力发展农民专业合作社，在全市建成300个规范合作社。加大财政支持力度，切实做好示范村、整治村建设，实施“一村一品、一乡一业”工程，引导农村发展支柱产业，加快中低产田改造，加强农村基础设施建设，加快农村现有公路改造升级，继续改造乡镇卫生院。建立健全农业技术推广服务体系，为建设新农村培养新型农民。加大扶贫开发力度，积极推进贫困村新农村建设试点工作。

　　四、突出抓好八项重点工程，强力推进重点项目建设

　　继续坚持以实施跨越式发展八项重点工程为抓手，统一认识、加强领导，不断完善机制、加强协调，突出抓好对增强我市经济社会发展后劲具有战略意义的重大项目，力求取得突破性进展。调整项目投资重点，加快项目建设步伐。不断提高农村基础设施和农村结构调整项目投资比重，加快交通、能源、水利等基础产业发展，支持基础教育、公共卫生等社会事业加快发展。强化枢纽中心城市地位，加快郑西铁路客运专线建设，争取开工建设郑石、郑武铁路客运专线，抓好郑州黄河公铁两用大桥、郑州高速公路客运枢纽站、郑州铁路客运专线枢纽站、郑州火车站西出口改造等项目建设。推进拓普轧钢、赵家寨煤矿、南水北调中线工程郑州段等重大项目建设，做好国电荥阳2×60万千瓦等项目前期工作。加强交通运输体系建设，郑州至石人山、登封至禹州高速公路建成通车，完成登封铁路二期工程和新郑机场改扩建工程，加快郑州西绕城公路、郑开大道与京珠高速互通立交等项目建设，做好郑州城市轻轨和郑汴城际轨道交通前期工作。

　　五、加快城市建设步伐，提高城市管理水平

　　深入开展“城市管理年”活动，加快城市建设重点工程进度，完善城市基础设施，进一步优化城市人居环境。

　　继续抓好中心城区保护改造。以古都保护和建设宜居城市为重点，抓好商城遗址等文物古迹保护修缮工作。进一步完善城市基础设施，完成中州大道、中原路、花园路和省行政区道路等精品街区综合整治，完成秦岭路、郑新路南段、沈庄北路等续建项目，对郑邙公路、化工路等出入市口和城乡接合部道路进行改造；完善城市夜景照明系统，逐步形成片、线、点、环有机结合的城市夜景照明体系。优先发展城市公交，拓展公交线网覆盖面，搞好水电气暖供应，保障居民生活需求。加快城市绿地系统建设和公园、街头游园的升级改造。

　　加快郑东新区建设。今年是郑东新区“五年成规模”的最后一年，要对照东区建设目标，进一步加快项目建设进度，突出抓好中央商务区、龙湖南区建设，完成河南艺术中心建设，加快建设会展宾馆和商业步行街建设。加快学校、医院、商业网点及基础配套设施建设，完善功能，提升品位，不断聚集东区人气。加大金融机构、企业总部引进力度，打造中央商务区金融中心和企业总部基地，吸引国内外品牌企业竞相入驻。完善东区市政管理体制，推行精细化管理。

　　带动中小城市和小城镇建设。加大卫星城市规划和建设力度，完善城市基础设施，不断增强服务功能，逐步建成与中心城区规划上衔接、功能上对接、产业上联接的中小城市群，努力构建大郑州都市圈。抓住郑汴一体化给中牟发展带来的机遇，充分发挥中牟在郑汴一体化发展中的战略支撑作用，加快推进中牟工业化、城市化进程。搞好总体规划和土地利用规划，加大资金、政策扶持力度，推进市级重点镇建设。

　　全面提高城市管理水平。深化城市管理体制改革，实行市、区双重管理体制，探索建立现代化的“大城管”管理体系，创新城市管理体制，制订和完善行业管理规范、标准，实现城市管理的规范化、制度化、法制化。深入开展爱国卫生运动，进一步巩固创卫成果，逐步建立市容环卫管理的长效机制。加快推进数字化城市管理系统建设，确保年内通过建设部验收。提高城市交通管理水平，逐步解决交通拥堵问题。将出入市口纳入城区统一管理，积极推进城中村、铁路沿线、老居民区、城郊接合部等区域的改造整治，深入开展户外广告、洗车行业、出租汽车行业专项治理活动，加强停车场建设和管理，有效治理城市环境中存在的脏、乱、差等突出问题。

　　六、发挥文化资源优势，加快推进文化建设

　　大力发展文化事业。加大对公益性文化事业投入，加强重大文化基础设施建设。启动商城遗址保护工程及商都文化苑建设项目，抓好郑州歌舞剧院、郑州图书馆新馆等项目建设。继续实施文化精品工程，认真组织《洛神》等一批文艺精品的生产创作。建立健全公共文化服务体系，加强县级文化馆、图书馆和乡镇文化站、新农村书屋建设，继续实施文化信息资源共享和农村电影放映工程。振兴传统文化产业，大力发展新兴文化产业，放开发展社会文化产业。整合古都文化资源，加强重点文物和非物质文化遗产保护、开发和利用，做好嵩山古建筑群申报世界文化遗产工作。精心筹备，办好丁亥年黄帝故里拜祖大典。着力壮大出版影视、广告会展、文化创意和动漫制作等产业，建设一批文化产业基地和特色文化产业园区，培育一批知名文化品牌。强化文化市场监管，深入开展“扫黄打非”斗争，加强知识产权保护。继续深化文化体制改革。抓好文化体制改革方案和配套政策的落实，深化文化事业单位内部人事、收入分配和社会保障制度改革，加快郑州豫剧院和郑州曲剧院合并，组建郑州戏曲艺术剧院，鼓励和引导非公有资本进入文化产业领域。加强和谐文化建设，大力弘扬以爱国主义为核心的民族精神和以改革创新为核心的时代精神，树立知荣辱、讲正气、促和谐的良好风尚。加强社会公德、职业道德、家庭美德教育，深入开展“道德规范进万家、诚实守信万人行”活动，办好第四届中国公民道德论坛。以创建全国文明城市为目标，广泛开展文明行业、文明单位和文明村户创建活动。

　　七、加强资源节约和环境保护，建设资源节约型和环境友好型社会

　　建立健全节能降耗目标责任制，加强对高耗能行业和重点耗能企业的监测管理，严格行业准入门槛，实行项目合理用能审查制度。加快推进节能技术进步，发展可再生能源和新能源。积极开展循环经济试点，出台加快发展循环经济的政策措施。整顿规范矿产资源开发秩序，提高矿产资源综合利用率。加强土地节约集约利用。严格控制新增建设用地规模，严格执行新的土地有偿使用费标准，严格实行土地出让制度。在土地供应上严把投资强度关，在工业聚集区大力推广使用多层标准厂房。继续推进土地“三项整治”，盘活存量建设用地。

　　全面落实环境影响评价制度、污染物排放总量控制制度。坚持行业和区域并重，对煤炭、电力、水泥、化工等重污染行业进行综合整治，有效减少主要污染物排放总量，继续开展区域流域环境综合整治。加快城区河道生态水系建设，加大金水河、熊儿河、东风渠截污和整治力度，实现市区段河渠水通、水清、水美的目标。加快王新庄污水处理厂脱氮设施、马头岗污水处理厂、中牟县污水处理厂建设步伐，开工建设五龙口污水处理厂二期扩建工程。继续开展城区环境综合整治，逐步拆除城区10蒸吨以下燃煤大锅炉。加快森林生态城建设，实施黄河湿地恢复与保护工程。强力推进创模工作，国家环保模范城市和全国绿化模范城市力争年内创建成功。

　　八、继续深化体制改革，不断提高对外开放水平

　　继续深化国有企业改革，重点抓好中原制药厂、郑州百文等企业的资产重组和改制工作。基本完成市直单位所属企业改革任务。大力发展非公有制经济，引导鼓励非公有制企业参与国企改革，吸引民间资本进入部分垄断行业。整合金融资源，加快组建金融控股集团等融资公司。积极推进投融资体制改革，拓宽投融资渠道。深化农村综合配套改革，深化和完善乡镇机构改革、农村义务教育改革，加快推进县乡财政管理体制改革，认真落实减轻农民负担的各项政策。扎实推进事业单位人事制度改革和文化、科技、医疗卫生等领域的各项改革。

　　加快发展开放型经济，不断提高招商引资的质量和水平。进一步修订完善发展开放型经济奖励政策，充分调动社会各界参与招商引资的积极性。改进招商方式，推进职业化招商。突出招商重点，引导和鼓励国内外资金投向我市高新技术产业、先进制造业、现代服务业、现代农业和环保产业。不断优化投资环境，切实保护外来投资者的合法权益。大力优化出口商品结构，提高出口产品附加值。大力推进食品和绿色农产品出口，进一步扩大农业对外开放。全面实施“走出去”战略，支持有条件的企业开展境外投资。大力发展对外劳务合作，继续加强区域经济协作与联络。充分发挥高新技术产业开发区、经济技术开发区和出口加工区在我市对外贸易和招商引资中的主平台作用。

　　九、统筹经济社会协调发展，努力构建和谐社会

　　把扩大就业放在政府工作的突出位置，努力实现经济发展和扩大就业良性互动。重点做好下岗失业人员再就业工作，高度重视“零就业”家庭和就业困难人员就业，加强对大学毕业生就业的指导和服务。加快农村人力资源中心市场建设，完善各级农村人力资源市场服务功能。逐步建立农民工培训基地，加大对农村剩余劳动力职业技能培训力度，提高转移就业能力。进一步加大农民工工资清欠力度，有效维护农民工合法权益。

　　不断完善社会保障制度，切实保障人民群众基本生活。完善企业职工基本养老保险制度，完善城镇职工基本医疗保险制度。扩大城乡低保覆盖面，做到动态管理下的应保尽保。建立多种形式的农村养老保险制度，进一步完善新型农村合作医疗制度。推进失业、工伤、生育保险制度建设。加大对困难群体的救助救济力度，大力发展社会福利和慈善事业。

　　大力实施科教兴郑和人才强市战略，积极发展各项社会事业。进一步推进科技自主创新工程建设，加强知识产权保护。坚持教育优先发展，推动公共教育资源向农村和薄弱学校倾斜，促进教育公平。整合教育资源，调整学校布局和结构，着力解决好入学难问题。加快发展职业教育，规划建设职业教育基地，争创1所国家示范性高等职业院校。以创新型高层次人才为重点，加强各类紧缺人才的培养、选拔和引进。加快建立覆盖城乡居民的基本卫生保健制度，重点加强农村卫生服务网络和新型城市卫生服务体系建设。加强对严重危害人民群众健康的重大传染病及慢性非传染性疾病的预防控制工作。加快市疾病预防控制中心、市七院迁建和市六院呼吸道病房楼建设，支持县级医院、妇幼保健院、中医院改造。整顿规范食品、药品市场秩序，切实抓好食品放心工程，加强食品、药品、餐饮卫生监管，保障人民群众饮食用药安全。健全新型社区管理和服务体制，完善社区卫生服务站，发挥社会组织在社区建设中的积极作用，争创全国和谐社区建设示范市。加强城乡体育设施建设，广泛开展全民健身活动，提高人民群众健康水平；提高竞技体育水平，力争在第六届全国城市运动会上取得好成绩。建立和完善计划生育工作新机制，继续稳定低生育水平，统筹解决出生人口性别比偏高、流动人口管理与服务等问题。

　　进一步落实信访工作责任，认真解决群众普遍关注的热点、难点问题，及时排查和化解各种社会矛盾，妥善处置群体性事件。健全应急预案体系，加强应急管理机构和队伍建设，提高危机管理和抗风险能力。坚持依法治市，深入开展“五五”普法。继续开展平安创建活动，严厉打击各类违法犯罪，完善社会治安防控体系。完善安全生产体制机制，坚决遏制重特大事故的发生，保障人民群众生命财产安全。

　　全面贯彻党的民族宗教政策，巩固平等、团结、互助、和谐的民族关系，促进各民族和睦相处，促进宗教和谐。认真抓好民兵预备役工作，深入开展国防教育和双拥共建活动，积极支持部队和国防建设，再创“全国双拥模范城”。继续发挥工会、共青团、妇联的桥梁和纽带作用。继续做好统计、审计、外事、侨务、对台事务、社会科学、地方志、档案管理、人防、气象和地震监测等各项工作。

　　十、坚持为民谋利，认真办好“十件实事”

　　通过广泛征集社会各界意见，确定了2007年为群众办好的“十件实事”：一是继续实施对种粮农民直接补贴、农业生产资料综合直补、优质专用小麦良种补贴、专用玉米良种补贴、农村劳动力转移培训补贴、测土配方施肥补贴、农机具购置补贴，确保补贴总金额高于上年。二是改善农村居民生活条件，解决20万人安全饮水问题，新建沼气池5万座、集雨水窖1万个，实现1万人脱贫。三是免除农村义务教育阶段学生学杂费、书作费，补助贫困寄宿生生活费，为全市农村义务教育阶段学校更新配置课桌凳16万套。四是全市新增城镇就业再就业12万人，农村劳动力转移就业12万人，及时解决“零就业”家庭就业问题。五是完成全市乡镇卫生院基础设施改造，建设30个社区卫生服务中心，推行城镇居民医疗保险制度，为全市1万名白内障患者免费实施复明手术。六是进一步扩大农村广播电视和信息网络覆盖面，实现全市无线数字电视信号全覆盖，新建100个社区文化活动中心。七是全市新建、扩建改造敬老院25所，农村五保户集中供养率提高到40%。八是完成森林生态城工程造林10万亩，中心城区新增绿地200万平方米，市区空气质量二级以上天数不低于285天。九是新开工经济适用住房70万平方米，竣工70万平方米，市区廉租住房覆盖率达到符合条件“双困家庭”100%。十是扩大城乡公共交通覆盖面，新改建农村公路1000公里。

　　切实加强政府自身建设不断推进政府管理创新

　　更好地贯彻落实科学发展观，适应发展社会主义市场经济和构建社会主义和谐社会的要求，实现郑州经济社会跨越式发展，必须深刻认识推进政府自身建设和管理创新的重要性和紧迫性，努力建设服务型政府、法治政府、责任政府和效能政府。

　　(一)加快职能转变，提高社会管理和公共服务水平。全面履行政府职能，在更好地履行经济调节和市场监管职能的同时，切实加强社会管理和公共服务职能。创新经济管理制度和方式，更多地运用经济手段和法律手段调节经济活动。继续推进政企分开，把经济工作的重点放在服务各类市场主体和创造良好发展环境上。深化行政审批制度改革，进一步减少和规范行政审批事项。按照建设服务型政府的要求，创新公共服务体制，改进公共服务方式，以发展社会事业和解决民生问题为重点，着力解决就业、就学、就医、社会保障、社会治安、安全生产、环境保护等人民群众最关心的利益问题，优化公共资源配置，加强公共设施建设，完善社会管理制度，提高社会管理水平，为全市人民提供更多更好的公共服务。

　　(二)坚持科学民主决策，不断提高决策水平。合理界定政府决策权限，进一步健全重大事项集体决策、专家咨询、社会公示与听证、决策评估等制度。凡是涉及经济社会发展中的重大决策，必须坚持调查研究和集体决策制度，并充分听取社会各界的意见。凡是与人民群众利益密切相关的重大事项，必须进行社会公示或者听证。各项决策都要尽可能符合实际，符合群众的意愿，有效避免决策失误。建立健全决策反馈纠偏机制和决策责任追究制度，对决策失误造成重大损失的，必须追究责任。

　　(三)切实依法行政，增强政府执行力和公信力。认真落实监督法，主动接受同级人大及其常委会的法律监督、工作监督，自觉接受政协的民主监督，虚心听取各民主党派、工商联和无党派人士的意见和建议。不断强化社会监督，依法保障人民群众实施监督的权利，高度重视舆论监督，强化政府内部的专门监督。严格依照法定权限和程序行使权力、履行职责，切实将政府管理经济社会行为纳入依法运转的轨道，做到有法必依、执法必严、违法必究。进一步明确行政执法权限，减少行政执法层级，完善执法程序，提高执法水平。完善行政复议、行政赔偿和补偿等制度，加大行政综合执法改革力度，坚决克服多头执法、执法不公甚至执法违法等现象，努力维护社会公正，增强政府的执行力和公信力。

　　(四)加强政风建设，着力提高行政效能。牢固树立为人民服务的宗旨，切实做到为民、务实、清廉，讲正气、树新风。大兴求真务实之风，坚持深入实际，调查研究，着力解决影响改革发展稳定的突出问题，切实维护群众利益。大力弘扬艰苦奋斗和勤俭节约精神，坚决反对拜金主义、享乐主义和奢靡之风。严格财政支出管理，严格控制公费出国出境和境外培训，严格规范公务接待行为。加强政府廉政建设，强化权力运行的制约和监督，加大商业贿赂专项治理工作力度。全面推进政务公开，不断扩大政务公开的范围和层次，不断提高电子政务运行水平，继续完善政府新闻发言人制度。强化行政问责制度，充分发挥绩效评估的导向作用和激励约束作用，不断提高政府效能，加强目标管理和督查，确保各项工作的落实。

　　各位代表，全面完成今年的各项目标和任务，实现我市经济社会跨越式发展，时不我待。让我们紧密团结在以胡锦涛同志为总书记的党中央周围，高举邓小平理论和“三个代表”重要思想伟大旗帜，在省委、省政府和市委的正确领导下，紧紧依靠全市人民，进一步解放思想，振奋精神，锐意进取，真抓实干，为实现经济社会又好又快发展，为实现中原崛起而努力奋斗！
